# Intra- and Interspecies Differences of Two *Cecropia* Species from Tabasco, Mexico, Determined through the Metabolic Analysis and ^1^H-NMR-Based Fingerprinting of Hydroalcoholic Extracts

**DOI:** 10.3390/plants12132440

**Published:** 2023-06-25

**Authors:** Eric Jaziel Medrano-Sánchez, Gloria Ivonne Hernández-Bolio, Carlos Ernesto Lobato-García, Manasés González-Cortazar, Mayra Antunez-Mojica, Ammy Joana Gallegos-García, Cristian Octavio Barredo-Hernández, Ricardo López-Rodríguez, Nelly Cristina Aguilar-Sánchez, Abraham Gómez-Rivera

**Affiliations:** 1 División Académica de Ciencias Básicas, Universidad Juárez Autónoma de Tabasco, Carretera Cunduacán-Jalpa Km. 0.5, Cunduacán 86690, Tabasco, Mexico; ericsanz123@gmail.com (E.J.M.-S.); carlos.lobato@ujat.mx (C.E.L.-G.); cristian_ba99@hotmail.com (C.O.B.-H.); ricardo.lopezr@ujat.mx (R.L.-R.); 2 Departamento de Física Aplicada, Centro de Investigación y Estudios Avanzados del Instituto Politécnico Nacional, Unidad Mérida, Antigua Carretera a Progreso Km. 6, Mérida 97310, Yucatán, Mexico; 3 Centro de Investigación Biomédica del Sur, Instituto Mexicano del Seguro Social, Argentina No. 1, Col. Centro, Xochitepec 62790, Morelos, Mexico; 4 CONACYT-Centro de Investigaciones Químicas-IICBA, Universidad Autónoma del Estado de Morelos, Avenida Universidad 1001, Chamilpa, Cuernavaca 62209, Morelos, Mexico; myam@uaem.mx; 5 División de Ciencias Básicas e Ingeniería, Universidad Popular de la Chontalpa-Carretera Cárdenas-Huimanguillo Km 2 S/N, Ranchería, Invitab Paso y Playa, Heroica, Cárdenas 86556, Tabasco, Mexico; joana90102010@gmail.com; 6 División Académica Multidisciplinaria de Jalpa de Méndez, Universidad Juárez Autónoma de Tabasco, Carretera Estatal Libre Villahermosa Comalcalco Km 27 S/N, Ranchería Rivera Alta, Jalpa de Méndez 86205, Tabasco, Mexico; nelly.aguilar@ujat.mx

**Keywords:** *Cecropia peltata*, *Cecropia obtusifolia*, metabolomic profile, chemical markers

## Abstract

The genus *Cecropia* is used in the traditional medicine of Tabasco, Mexico, in diabetes and hypertension treatments, mainly without distinction of the species. This contribution aimed to carry out the metabolic analysis and Proton Nuclear Magnetic Resonance (^1^H-NMR) spectroscopy-based fingerprinting of the hydroalcoholic leaf extracts of *Cecropia peltata* (**Cp**) and *Cecropia obtusifolia* (**Co**) collected in five sub-regions of the State of Tabasco (**Cp1**, “Centro”; **Cp2**, “Chontalpa”; **Cp3**, “Pantanos”; **Cp4**, “Ríos” and **Co5**, “Sierra”). Firstly, the extracts were evaluated for their Total Phenol Content (TPC) and Total Flavonoid Content (TFC) by spectrophotometric methods. In addition, metabolic analysis was performed using High-Performance Liquid Chromatography with Diode-Array Detection HPLC–DAD, which allowed the quantification of the chemical markers: chlorogenic acid, isoorientin, and orientin, as well as a vitexin analog. Finally, metabolomic analysis was carried out based on the ^1^H-NMR spectra. The **Cp4** extract (*C. peltata* from the “Ríos” sub-region) presented the highest values of TPC (155 ± 9.1 mg GAE/g E) and TFC (724 ± 22.2 mg RE/g E). The metabolic analysis was similar among the five samples; the highest concentrations of the four chemical markers were found in **Cp3** (*C. peltata* from the “Pantanos” sub-region) for chlorogenic acid (39.8 ± 2.3 mg/g) and isoorientin (51.5 ± 2.9 mg/g), in **Cp4** for orientin (49.9 ± 0.6 mg/g), and in **Cp2** (*C. peltata* from the “Chontalpa” sub-region) for the vitexin analog (6.2 ± 0.2 mg/g). The metabolic analysis and the ^1^H-NMR fingerprint analysis showed intraspecies differences among the *C. peltata* samples and interspecies between *C. peltata* and *C. obtusifolia*, which were attributed to variations in the metabolite groups as well as in the proportion of sugars such as glucose and xylose.

## 1. Introduction

Plant metabolomics is a field of increasing interest due to different applications, such as knowing the impact that different environmental factors have on metabolism, determining the tolerance to stress by abiotic and biotic agents, as well as determining the complexity of the metabolic content between species of the same genus [1,2,3]. A first approach to determine the metabolic profile of a plant species is to use spectrophotometric and chromatographic techniques that allow the quantification of groups of metabolites as well as the identification of chemical markers of interest [4,5]. In addition, Hydrogen Nuclear Magnetic Resonance (^1^H-NMR) is one of the main analytical tools used to perform metabolomic studies, as this technique shows a broad overview of the metabolic content in plant extracts, with the advantage of detecting metabolites of various polarities, easily allowing their quantification. Furthermore, NMR provides enough information to carry out studies of structural elucidation without requiring derivatization processes [6]. The ease of processing the ^1^H-NMR spectra using several computer programs, as well as the managing, interpretation, and statistical analysis of the data, makes this technique a fast and convenient tool for discriminating species based on their metabolic content [7,8]. For instance, a recent study showed the effectiveness of using ^1^H-NMR to evaluate differences in chemical markers related to the different growth stages of *Peganum harmala* L. [9].

In this sense, the genus *Cecropia* constitutes a group of plants that presents challenges in defining of its metabolic profile. This genus is distributed from the tropical and subtropical jungle of Mexico to South America and is made up of 61 species—most of them are traditionally named “guarumo” or variants of this name such as “guarumbo” or “yarumbo”; in the Yokot’an language spoken in the state of Tabasco, the word “k’olok’” is employed [10,11]. It is noteworthy that folk names do not distinguish between species of this genus, which implies challenges in the systematic knowledge of their use in traditional medicine. Different reports on *Cecropia* spp. emphasize the use of these plants in diabetes [12,13] and hypertension [14,15] treatments, as well as in the alleviation of inflammation and pain [16]. In addition, antimicrobial [17] and antimalarial properties have also been reported [18]. In Tabasco State, Mexico, *C. peltata* and *C. obtusifolia* are the two species with the greatest distribution and use [19]. The coexistence of both species with the same popular name can lead to potentially inappropriate use derived from the null differentiation between them, with the concomitant risk that the substitution of one plant species for another may have implications for the therapeutic effect that is being sought, including potentially adverse health reactions. In this sense, the evaluation of the hypoglycemic effect of methanolic extracts from the leaves of *C. peltata* and *C. obtusifolia* demonstrated a greater effectiveness for the first species, attributed to a higher concentration of chlorogenic acid, which is the main metabolite related to this activity [20]. Furthermore, it is important to indicate that the metabolic content of a plant may present differences depending on the region where it is collected [21,22,23].

The state of Tabasco is located in the southeast of Mexico and is officially divided into five sub-regions (“Centro”, “Chontalpa”, “Pantanos”, “Ríos”, and “Sierra”) that are delimited by geographical, cultural, and productive aspects [24]. In these sub-regions, inhabitants traditionally use the leaves of both *Cecropia* species (*C. peltata* and *C. obtusifolia*) as an infusion for the treatment of diabetes [10,25]. However, reports concerning the phytochemical and/or metabolomic analyses of these *Cecropia* species are scarce. In order to promote the rational use of extracts and herbal products derived from these plants, it is necessary to generate information that allows an adequate recognition of their phytochemical contents. In this sense, the objective of this study was to characterize the metabolic composition of the hydroalcoholic extracts from the leaves of *C. peltata* and *C. obtusifolia* collected in each of the five sub-regions of the state of Tabasco, emphasizing the study of the quantification of chlorogenic acid, isoorientin, orientin, and a vitexin analog, which are well recognized as chemical markers for this genus [26]. Additionally, a multivariate analytical approach was applied by the assessment of ^1^H-NMR fingerprinting of the aforementioned extracts in order to have a comprehensive view of the chemical composition of these species.

## 2. Results and Discussion

### 2.1. Total Phenol Content (TPC) and Total Flavonoid Content (TFC) of Hydroalcoholic Extracts from Cecropia Species

**Figure 1:**
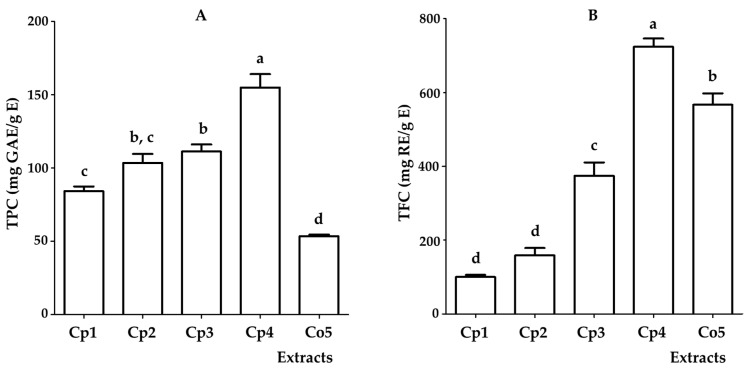
Comparison of TPC (**A**) and TFC (**B**) in the hydroalcoholic extracts of *C. peltata* (**Cp1–Cp4**) and *C. obtusifolia* (**Co5**). TPC: Total phenol content; mg GAE/g E: Gallic acid equivalents per gram of extract. TFC: Total flavonoid content; mg RE/g E: rutin equivalents per gram of extract. The data represent the mean ± standard error (*n* = 3) of each extract. Statistical significance was determined using analysis of variance (ANOVA) followed by a post hoc Tukey test. Bars with the same letter (a–d) represent no significant difference at *p* < 0.05.

The quantification of TPC and TFC in the five samples analyzed is shown in Figure 1. The sample identified as *C. peltata* from the “Ríos” sub-region (**Cp4)** showed the highest concentration of both groups of metabolites. The TPC from extract **Cp4** (155 ± 9.1 mg GAE/g E) presented significant differences with respect to the other samples, whereas **Cp2** from the “Chontalpa” sub-region and **Cp3** from the “Pantanos” sub-region did not show significant differences in the TPC values (103.4 ± 6.3 and 111.3 ± 4.8 mg GAE/g E, respectively). It is noteworthy that **Cp2** also did not show significant differences in the content of phenolic compounds when compared to **Cp1** from the “Centro” sub-region (84.1 ± 3.3 mg GAE/g E). It should also be noted that the extract of *C. obtusifolia* from the “Sierra” sub-region (**Co5**) presented the lowest concentration of TPC (53.5 ± 1.1 mg GAE/g E) and exhibited a significant difference with respect to the *C. peltata* samples. Regarding the quantification of TFC, **Cp4** showed a content of 724 ± 22.2 mg RE/g E, with a significant difference compared to the other extracts. The extracts **Co5** (567.1 ± 30.5 mg RE/g E) and **Cp3** (374.5 ± 36.1 mg RE/g E) were the second and third samples, respectively, with the highest content of flavonoids, with a significant difference between them. On the other hand, the **Cp1** and **Cp2** samples presented the lowest TFC (100.1 ± 5.5 and 158.8 ± 19.6 mg RE/g E, respectively), with no significant difference between them.

These results contrast with those reported by Rivera-Mondragón et al., 2019, concerning the TPC and TFC contents of several species of *Cecropia* in Panama; *C. obtusifolia* had the highest content of these metabolites [27]. However, in the present study, the *C. peltata* samples (**Cp1–Cp4**) presented higher levels of TPC in contrast to the *C. obtusifolia* sample (**Co5**), which presented the lowest content of this group of metabolites. Similarly, when comparing the values of TFC, an extract of *C. peltata* (**Cp4**) was the one with the highest concentration of flavonoids, followed by the *C. obtusifolia* sample (**Co5**).

### 2.2. Preliminary HPLC Analysis of Hydroalcoholic Extracts from Cecropia Species

**Figure 2:**
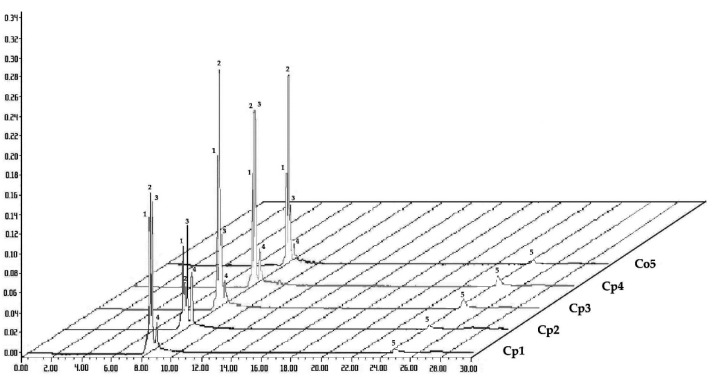
HPLC chromatograms of the hydroalcoholic extracts of *C. peltata* (**Cp1–Cp4**) and *C. obtusifolia* (**Co5**). The peaks are numbered in ascending order according to their retention times (λ = 330 nm). **1**, chlorogenic acid; **2**, isoorientin; **3**, orientin; **4**, vitexin analog; **5** cathequin analog.

The analysis of the HPLC chromatograms obtained from the hydroalcoholic extracts of the *Cecropia* spp. (Figures S1–S5) allowed the identification of five peaks with a retention time (*t*_R_) ranging from 8.5 to 24.9 min (Figure 2). They were characterized as follows: Peak **1** with a *t*_R_ of 8.53 min showed absorption bands at λmax = 220.4 and 327.6 nm; these parameters coincided with those of the chlorogenic acid standard analyzed under the same experimental conditions. Likewise, peaks **2** and **3** were identified as isoorientin (*t*_R_ of 8.65 min) and orientin (*t*_R_ of 8.78 min); the absorption bands of both peaks at λmax = 208.7, 269.8, and 351.7 nm and at 215.7, 268.6, and 345.8 nm, respectively, coincided with the standards analyzed under the same experimental conditions. In the case of peak **4**, with a *t*_R_ of 9.06 min, the absorption bands at λmax = 215.7, 268.6, and 336.3 nm showed similarity with those reported for a flavone-type glycosylated compound that can be attributed to a vitexin analog that was previously reported in *Cecropia* spp. [27,28,29,30]. Additionally, the chromatograms showed peak **5**, with a *t*_R_ of 24.9 min and absorption bands at λmax = 209.9 and 279.3 nm, which exhibited similarity to an esterified catechin analog that has been reported for this genus [26]. It is worth noting that the results obtained are in good agreement with previous reports on the phytochemical content of this genus [31,32,33,34,35]. Likewise, this study highlights the presence of a similar metabolic profile for these four metabolites in the extracts analyzed from *C. peltata* and *C. obtusifolia*.

**Figure 3:**
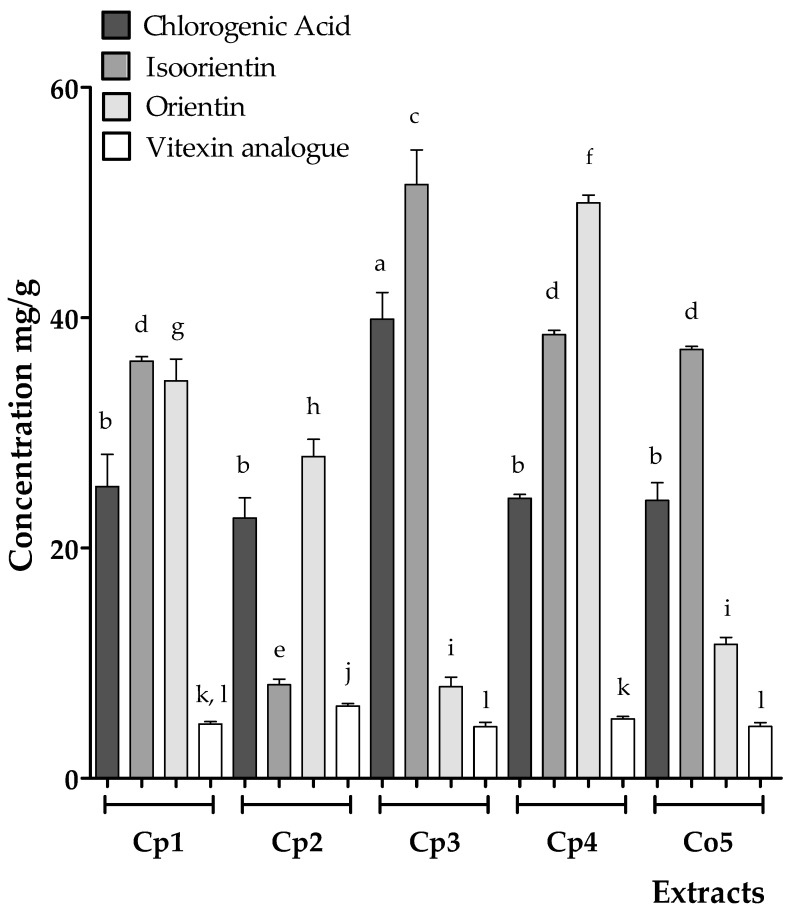
Concentration (mg/g) of chlorogenic acid, isoorientin, orientin, and the vitexin analog in the hydroalcoholic extracts of *C. peltata* (**Cp1–Cp4**) and *C. obtusifolia* (**Co5**). The data represent the mean ± standard error (*n* = 3) for each extract. Statistical significance was determined using analysis of variance (ANOVA) followed by a post hoc Tukey test. Bars with the same letter (a–l) represent no significant difference at *p* < 0.05.

Chlorogenic acid, isoorientin, orientin, and the vitexin analog have been established as chemical markers in quality control for the medicinal use of *Cecropia* species [26,30]. The quantification of these metabolites by HPLC are summarized in Figure 3. The **Cp3** sample presented the highest concentrations of chlorogenic acid and isoorientin: 39.8 ± 2.3 mg/g and 51.5 ± 2.9 mg/g, respectively, whereas two extracts of *C. peltata* (**Cp1** and **Cp4**) and the extract of *C. obtusifolia* (**Co5**), did not show significant differences for both chemical markers: chlorogenic acid (25.3 ± 2.8, 24.3 ± 0.3, and 24.1 ± 1.5 mg/g, respectively) and isoorientin (34.6 ± 0.3, 38.5 ± 0.3, and 37.2 ± 1.5 mg/g, respectively). However, a particular case was detected when analyzing the extract **Cp2**; although it showed similarity in its chlorogenic acid content (22.61 ± 1.7 mg/g) with respect to **Cp1**, **Cp4**, and **Co5**, it was the sample with the lowest concentration of isoorientin (8.1 ± 0.4 mg/g), with a significant difference when compared with the other extracts. In the case of orientin, **Cp4** showed the highest content (49.9 ± 0.6 mg/g), followed by **Cp1** (34.5 ± 1.8 mg/g) and **Cp2** (27.9 ± 1.5 mg/g), with significant differences between them; likewise, **Cp3** and **Co5** exhibited the lowest concentrations (7.9 ± 0.8 and 11.6 ± 0.6 mg/g, respectively), with no significant difference between them. For the vitexin analog, the quantification was performed by employing the aglycone moiety apigenin, and the sample with the highest content was **Cp2** (6.2 ± 0.2 mg/g), which was significantly different from the other samples, followed by **Cp4** (5.1 ± 0.2 mg/g) and **Cp1** (4.7 ± 0.2 mg/g), which were not different. The samples with the lowest concentration of this compound were **Cp3** (4.4 ± 0.4 mg/g) and **Co5** (4.5 ± 0.3 mg/g); these values were significantly different from **Cp4**, but they were statistically similar to **Cp1**. Regarding the esterified catechin analog, the area under the curve obtained was unsuitable for quantification. These results are consistent with those of previous studies that showed regional variability in the concentration of these chemical markers, which may impact the use attributed to the species in traditional medicine [20,27,34].

The spectroscopic and chromatographic methods used for the quantification of TPC, TFC, and the total chemical marker content (TCMC) are not comparable to each other, as each one has particular scopes and limitations [36,37,38]. Therefore, it was not possible to establish a direct relationship between the TPC, TFC, and TCMC values. However, it was possible to observe a trend that marked differences both within and between species, where *C. peltata* (**Cp4**) from the “Ríos” sub-region differed from the other samples of the same species (**Cp1**, **Cp2**, and **Cp3**), with higher values of TPC, TFC, and TCMC. On the other hand, *C. obtusifolia* (**Co5**) showed a statistically significant variation in TPC and TFC with respect to all *C. peltata* samples and was similar only to **Cp2** in TCMC (Table 1).

### 2.3. Multivariate Analysis of ^1^H-NMR Profiles

**Figure 4:**
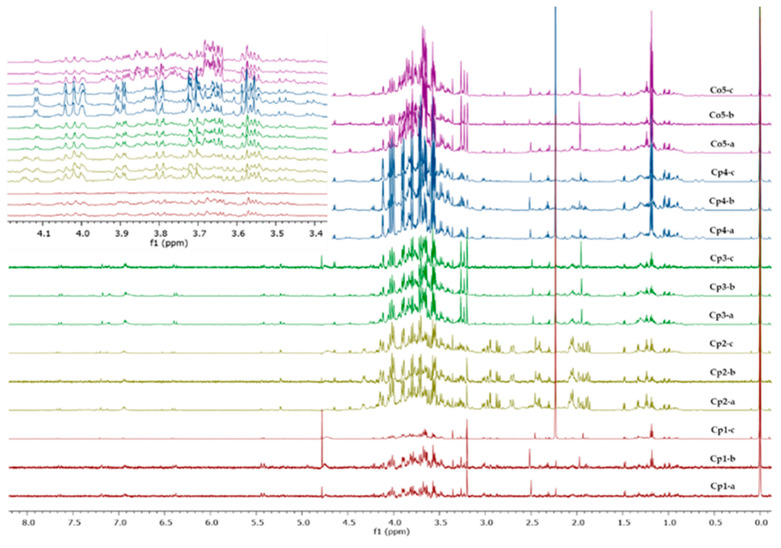
^1^H-NMR profiles (D_2_O, 600 MHz; region δ 8.00 to 0.00 ppm) of the hydroalcoholic extracts of *C. peltata* (**Cp1–Cp4**) and *C. obtusifolia* (**Co5**); a–c corresponds to triplicates of each sample. Inserted spectra show the region from δ 3.40 to δ 4.20 ppm with the characteristic signals of sugars.

The ^1^H-NMR metabolic profiles of each of the hydroalcoholic extracts of *Cecropia* spp. are shown in Figure 4.

**Figure 5:**
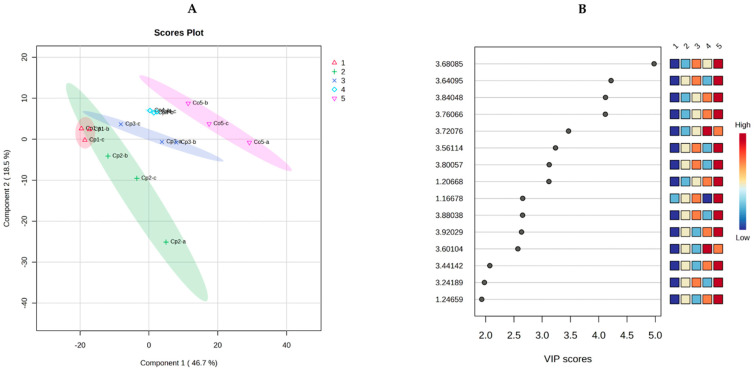
Principal component analysis score plot (PC1 vs. PC2, 65.2% explained variance); (**A**) ^1^H-NMR profiles of the hydroalcoholic extracts of *C. peltata* (**Cp1–Cp4**) and *C. obtusifolia* (**Co5**); a–c correspond to triplicates of each sample. (**B**) Variable Importance in Projection (VIP) of the defined metabolites of the ^1^H-NMR profiles of the hydroalcoholic extracts from *Cecropia* species.

The ^1^H-NMR data, used in the principal component analysis and the PCA score graph (PC1 vs. PC2), were obtained with 65.2% explained variance, as shown in Figure 5A. This allowed the identification of differences in the metabolic content for both intra- and interspecies. In the first case, **Cp4** represented an independent group differentiated from the other three samples of the same species, while, **Cp1**, **Cp2**, and **Cp3** belonged to the same group, with **Cp1** and **Cp3** being different from each other but similar to **Cp2**. On the other hand, the interspecies differences were very noticeable, as the sample of *C. obtusifolia* (**Co5**) represented an independent grouping with no affinity to *C. peltata*.

The PLS-DA generated the VIP values (Figure 5B), where the chemical shifts with the highest score were located mainly in the region of methines of oxygenated bases (δ 3.24–δ 3.92 ppm) that can be attributed, according to the literature [39,40], to the presence of sugars such as glucose (δ 3.68, δ 3.76, and δ 3.88 ppm) and xylose (δ 3.60 and δ 3.72 ppm), which have been previously reported in *Cecropia* species [27]. These results are consistent with the intraspecies differences observed in the PCA, as the independent group of *C. peltata* from the “Ríos” sub-region (**Cp4**) obtained a higher score in chemical shifts attributed to xylose than **Cp1**, **Cp2**, and **Cp3**. By contrast, the intraspecies differences observed in the PCA between **Cp1** and **Cp3** can be associated with a higher score, in the second sample, for the chemical shifts attributed to glucose and xylose. The interspecies differences were also corroborated, as *C. obtusifolia,* which represented an independent group in the PCA, was the sample with the highest score for the chemical shifts attributed to both sugars.

It is noteworthy that the analytical methods applied in this contribution revealed intra- and interspecies differences in the *Cecropia* samples studied, by quantifying specific groups of metabolites (TPC, TFC, and TCMC) and by analyzing the pattern in the ^1^H-NMR fingerprinting of whole extracts. The general tendencies that allowed the identification of the diversity between samples remained the same. These results highlight the importance of applying diverse analytical techniques in order to obtain a more comprehensive view of the metabolic differences that may be involved in the analysis of samples of the same species obtained from different sub-regions or samples of different species of the same genus.

Finally, it is important to highlight that the presence of polyphenols and flavonoids, as well as that of the four chemical markers, correlates with the use of these species in traditional medicine. However, the intra- and interspecies differences in the concentrations of these metabolites suggest the need to establish adequate conditions for the rational use of both species for the treatment of diabetes and hypertension, the main diseases for which *Cecropia* species are employed.

## 3. Materials and Methods

### 3.1. Plant Material and Extraction of Cecropia Species

**Figure 6:**
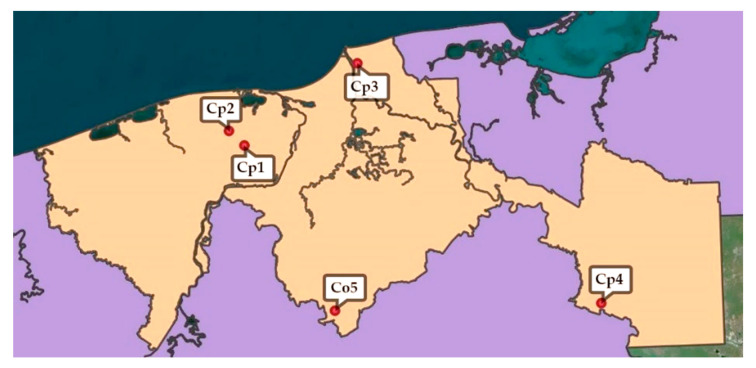
Collection points in Tabasco, Mexico, of leaves of *C. peltata* (**Cp1–Cp4**) and *C. obtusifolia* (**Co5**); map was made using QGIS^®^ v. 3.30.3 (Beaverton, OR, USA).

Mature specimens of *Cecropia* spp. (with a height greater than 2 m) [11] were identified at the five sampling points (Figure 6). The leaves of *Cecropia* spp. were collected in triplicate in the five sub-regions of the state of Tabasco, Mexico, in the periods of March–May and August 2021.

Table 2 shows the data corresponding to the geolocation (Google Maps^®^, Mountain View, CA, USA) of the points where each of the samples was collected. One specimen from each collection was deposited in the Herbarium of the Universidad Juárez Autónoma de Tabasco, Mexico.

The leaves were dried at room temperature in the dark for 72 h and ground (4–6 mm) using a Pulvex MP300. The dried plant materials (500 g each) were extracted by maceration with a mixture of reaction-grade solvents (ethanol:water 7:3 (2 L); Merck, Darmstadt, Germany); the maceration procedure was performed three times (24 h each) for each sample in order to ensure an exhaustive extraction. These extracts were filtered, concentrated in a rotary evaporator (Büchi R-300, Flawil, Switzerland), and then lyophilized (Freeze Dryer, Scientz-10N); a total of 15 extracts were obtained, and the labels for their identification and their average yield are shown in Table 3.

### 3.2. Total Phenol Content of the Hydroalcoholic Extracts of C. peltata and C. obtusifolia

The total phenol content was determined according to the Folin–Ciocalteu method described by Difonzo et al., 2022. The extract (10 mg) was properly diluted in 1 mL of MeOH (179337, Merck, Darmstadt, Germany); an aliquot of 100 µL was mixed with 250 µL of Folin–Ciocalteu reagent (47641, Sigma–Aldrich, St. Louis, MO, USA) and 1400 µL of H_2_O; this mixture was incubated for 3 min and subsequently, 250 µL of 20% of Na_2_CO_3_ (223530, Sigma–Aldrich, St. Louis, MO, USA) was added; the solution was incubated in the dark for 2 h at room temperature. The absorbance was measured at 760 nm using a spectrophotometer (GENESYS™ 10S UV–Visible Spectrophotometer, Thermo Scientific, Madison, WI, USA). A calibration curve was prepared with standard solutions of gallic acid (398225, Sigma–Aldrich, St. Louis, MO, USA) in MeOH (1.0, 2.0, 4.0, 6.0 and 7.0 µg/mL). The curve had an R^2^ of 0.9991. The results were expressed as mg of gallic acid equivalent per g of extract (mg GAE/g E). The analyses were carried out in triplicate [41].

### 3.3. Total Flavonoid Content of the Hydroalcoholic Extracts of C. peltata and C. obtusifolia

The total flavonoid content was measured by a colorimetric assay developed by Zhu et al., 2010. An aliquot of the diluted sample in MeOH (100 µL) was mixed with 1675 µL of H_2_O, and 75 µL of NaNO_2_ (5%, *w*/*w*; 221341, Sigma–Aldrich, St. Louis, MO, USA) was added to the flask. After 5 min, 150 µL of AlCl_3_ (10% *w*/*w*; 206911, Merck, Darmstadt, Germany) was incorporated, and the reaction was quenched 6 min later by the addition of 500 µL of NaOH (1 M; 221465, Merck, Darmstadt, Germany); the solution was incubated in the dark for 2 h at room temperature. The absorbance of the mixture was determined at 510 nm using a spectrophotometer (GENESYS™ 10S UV-Visible Spectrophotometers, Thermo Scientific, Madison, Wisconsin, USA). A calibration curve was prepared with rutin standard solutions (10.0, 20.0, 30.0, 40.0, and 50.0 µg/mL; 78095, Sigma–Aldrich, St. Louis, MO, USA). The calibration curve had an R^2^ of 0.9994. The results were expressed as mg of rutin equivalents per g of extract (mg RE/g E). The analyses were carried out in triplicate [37].

### 3.4. Preliminary HPLC Analysis and the Quantification of Chlorogenic Acid, Isoorientin, Orientin, and the Vitexin Analog in Hydroalcoholic Extracts of C. peltata and C. obtusifolia

The chromatographic analysis was performed as described by Gallegos-García et al., 2022. In general, it was performed using a Waters 2695 chromatograph with a Waters 2696 separation module and a photodiode array detector with Empower Pro software (Waters Corporation, Milford, MA, USA). A Supelcosil LC-F column (4.6 mm × 250 mm i.d., particle size 5 µm; Sigma–Aldrich, Bellefonte, PA, USA) was used. The mobile phase consisted of a 0.5% aqueous solution of trifluroacetic acid (302031, Sigma–Aldrich, St. Louis, MO, USA), as solvent A and acetonitrile (34851, Merck, Darmstadt, Germany) as solvent B, with the following gradient: 0–1 min, 0% of B; 2–3 min, 5% of B; 4–20 min, 30% of B; 21–23 min, 50% of B; 24–25 min, 80% of B; 26–27 min, 100% of B; 28–30 min, 0% of B; the flow rate was 0.9 mL/min. The sample volume was 10 µL of a methanolic solution of each extract (2 mg/mL) [42]. Likewise, a spectrophotometric scan was performed from 200 to 600 nm, and it was observed that by adjusting the detector to 330 nm, the distortion of the linear base decreased, and the peaks became more defined with a greater definition of the absorption bands corresponding to the identified compounds.

Quantification of the chlorogenic acid, isoorientin, orientin, and the vitexin analog was performed using calibration curves of external standards. Each calibration curve was constructed by injecting 50 µL of serial dilutions of pure chlorogenic acid (C3878, Sigma–Aldrich, St. Louis, MO, USA), isoorientin (78019, Sigma–Aldrich, St. Louis, MO, USA), and orientin (55736, Sigma–Aldrich, St. Louis, MO, USA); in the case of the vitexin analog, the quantification was performed with apigenin standard (42251, Sigma–Aldrich, St. Louis, MO, USA); all extracts and standards were dissolved in MeOH (12, 25, 50, 100, and 200 µg/mL). The calibration curves were based on peak areas of the HPLC chromatograms and had an R^2^ of 0.998, 0.999, 0.999, and 0.9998, respectively. The results are represented as mg per g of extract [20,43]. To visualize the differences in the content of chemical markers in the *Cecropia* species, the Total Chemical Marker Content (TCMC) was expressed as the sum of the concentrations of each chemical marker (chlorogenic acid, isoorientin, orientin, and the vitexin analog) in each sample.

### 3.5. ^1^H-NMR Fingerprinting and Multivariate Analysis of the Hydroalcoholic Extracts of C. peltata and C. obtusifolia

^1^H-NMR experiments were performed as described by Hernández-Bolio et al., 2021, with some modifications. The samples were processed at 25 °C on a Varian AR Premium Compact at 600 MHz. In brief, 10 mg of each extract was dissolved in 700 µL of D_2_O (0.05% TSP; 450510, Sigma–Aldrich, St. Louis, MO, USA) and transferred to a 5 mm NMR tube. The ^1^H-NMR spectra were recorded with the following parameters: pulse width: 5.9 µs, relaxation time: 1.0 s, number of transients: 64 with data collected from 65K data points. During the recycle delay, a pre-saturation sequence was employed to suppress the residual H_2_O signal with a selective low-power irradiation at the water resonance frequency. The resulting spectra were processed using MNova 12.0 (MestreLab Research S.L.) as described below. The phase was manually adjusted and the baseline was automatically corrected, a Gaussian function with a line-broadening factor of 0.3 Hz was applied, and the reference was set to sodium 3-trimethlysilyl [2, 2, 3, 3-d4] propionate (TSP; 0.0 ppm). After the spectra were stacked, the region from δ 4.6–4.8 corresponding to residual water was deleted to discard the effects of these intense signals in the multivariate analysis. Afterwards, the spectra were normalized to the TSP signal and divided into intervals of equal size of 0.04 ppm each to obtain a total of 301 regions. The multivariate data were analyzed using Principal Component Analysis (PCA) and Partial Least-Squares Discriminant Analysis (PLS-DA) by MetaboAnalyst Software version 5.0 (www.metaboanalyst.ca, Alberta, Canada; accessed on 26 January 2023). Those metabolites relative to the different species and/or influencing the discrimination of the samples were identified using Chenomx NMR suite software (v. 8.3, evaluation version, Chenomx Inc. Edmonton, Canada) [44].

### 3.6. Statistical Analysis

The total phenol, flavonoid, chlorogenic acid, isoorientin, orientin, vitexin analog, and total chemical marker contents were expressed as the mean ± standard error of the mean (SEM), and statistical significance was determined using analysis of variance (ANOVA) with a confidence level of 95% (* *p* ≤ 0.05) followed by the Tukey test. All statistics analyzed were performed using GraphPad Prism 5.0 (GraphPad Software, San Diego, CA, USA).

## 4. Conclusions

The hydroalcoholic extracts of the *Cecropia* species collected in the five sub-regions of the state of Tabasco, Mexico, presented both intra- and interspecies differences based on the content of the metabolic groups analyzed. *C. peltata* from the “Ríos” sub-region showed the highest values of TPC, TFC, and TCMC compared to the other samples of the same species; on the other hand, *C. obtusifolia* from the “Sierra” sub-region showed differences in TPC and TFC with respect to the *C. peltata* samples, but it was similar in TCMC to *C. peltata* from the “Chontalpa” sub-region. Likewise, a similar behavior was demonstrated in the multivariate analysis of the ^1^H-NMR-fingerprinting, in which the differences were based on the proportions of those signals attributed to sugar moieties present in the samples; *C. peltata* from the “Ríos” sub-region again showed a difference when compared to the other samples of the same species by presenting a higher proportion of signals attributed to xylose. By contrast, the *C. obtusifolia* sample was completely differentiated from all *C. peltata* samples, as it presented the highest proportion of signals attributed to glucose. It should be noted that the application of various analytical techniques found differences in the metabolic content of plants of the same species collected in different sub-regions, as well as in species belonging to the same genus. This report constitutes a first study, to our knowledge, among species of the genus *Cecropia* collected in the southeastern region of Mexico; therefore it contributes to the knowledge of the chemical contents of these plants. Finally, this work seeks to support the design of specific actions aimed at the rational use of herbal products derived from plants of this genus.

## Figures and Tables

**Table 1:** TPC, TFC, and TCMC in species of *Cecropia* *.

	Cp1	Cp2	Cp3	Cp4	Co5
TPC (mg GAE/g E)	84.1 ± 3.3 ^c^	103.4 ± 6.3 ^b,c^	111.3 ± 4.8 ^b^	155 ± 9.1 ^a^	53.5 ± 1.1 ^d^
TFC (mg RE/g E)	100.1 ± 5.5 ^d^	158.8 ± 19.6 ^d^	374.5 ± 36.1 ^c^	724 ± 22.2 ^a^	567.1 ± 30.5 ^b^
TCMC (mg/g)	100.8 ± 4.2 ^b^	64.9 ± 1.0 ^c^	103.9 ± 4.7 ^b^	118.0 ± 0.7 ^a^	77.4 ± 1.1 ^c^

* TPC: Total Phenol Content; TFC: Total Flavonoid Content and TCMC: Total Chemical Marker Content of the hydroalcoholic extracts of *C. peltata* (**Cp1–Cp4**) and *C. obtusifolia* (**Co5**). Statistical significance was determined using analysis of variance (ANOVA) followed by a post hoc Tukey test. Values with the same letter (a–d) represent no significant difference at *p* < 0.05, for the same assay.

**Table 2:** Geolocation of collection sites and taxonomic identification of *Cecropia* species.

Sub-Region	Municipality	Geographical Coordinates	Species	Voucher Number
“Centro”	Jalpa de Méndez	N 18°11′50.046″/W 93°10′15.2544″	*C. peltata*	36575
“Chontalpa”	Comalcalco	N 18°15′47.0916″/W 93°14′22.884″	*C. peltata*	36575
“Pantanos”	Centla	N 18°32′22.8084″/W 92°38′11.7852″	*C. peltata*	36666
“Ríos”	Tenosique	N 18°11′50.046″/W 93°10′15.2544″	*C. peltata*	36575
“Sierra”	Tacotalpa	N 17°27′28.2024″/W 92°46′46.2108	*C. obtusifolia*	36667

**Table 3:** Nomenclature and percentages for hydroalcoholic extracts obtained from *C. peltata* (**Cp**) and *C. obtusifolia* (**Co**).

Sub-Region	Label *	Average Yield %
“Centro”	Cp1a–c	9.3 ± 2.5
“Chontalpa”	Cp2a–c	10.5 ± 3.2
“Pantanos”	Cp3a–c	13.2 ± 2.6
“Ríos”	Cp4a–c	8.6 ± 1.8
“Sierra”	Co5a–c	10.5 ± 2.2

* a–c corresponds to triplicates of each sample.

## Data Availability

Data are contained within the article and Supplementary Material.

## Supplementary Materials

The following supporting information can be downloaded at https://www.mdpi.com/article/10.3390/plants12132440/s1, Figure S1: HPLC chromatogram and UV spectra of the main compounds of hydroalcoholic extracts of **Cp1**; Figure S2: HPLC chromatogram and UV spectra of the main compounds of hydroalcoholic extracts of **Cp2**; Figure S3: HPLC chromatogram and UV spectra of the main compounds of hydroalcoholic extracts of **Cp3**; Figure S4: HPLC chromatogram and UV spectra of the main compounds of hydroalcoholic extracts of **Cp4**; Figure S5: HPLC chromatogram and UV spectra of the main compounds of hydroalcoholic extracts of **Co5**.
