# Supplementary material for: Intra- and Interspecies Differences of Two Cecropia Species from Tabasco, Mexico, Determined through the Metabolic Analysis and 1H-NMR-Based Fingerprinting of Hydroalcoholic Extracts
Source: Plants (Basel). 2023 Jun 25;12(13):2440. doi: 10.3390/plants12132440 (PMC10346827; doi:10.3390/plants12132440)
Supplement: Supplementary file 1 [file plants-12-02440-s001.zip › plants-2432179-supplementary.pdf]

Intra- and Interspecies Differences of Two *Cecropia* Species from Tabasco, Mexico, Determined through Metabolic Analysis and ^{1}H-NMR-Based Fingerprinting of Hydroalcoholic Extracts

Eric Jaziel Medrano-Sánchez, Gloria Ivonne Hernández-Bolio, Carlos Ernesto Lobato-García, Manasés González-Cortazar, Mayra Antúnez-Mojica, Ammy Joana Gallegos-García, Cristian Octavio Barredo-Hernández, Ricardo López-Rodríguez, Nelly Cristina Aguilar-Sánchez and Abraham Gómez-Rivera.

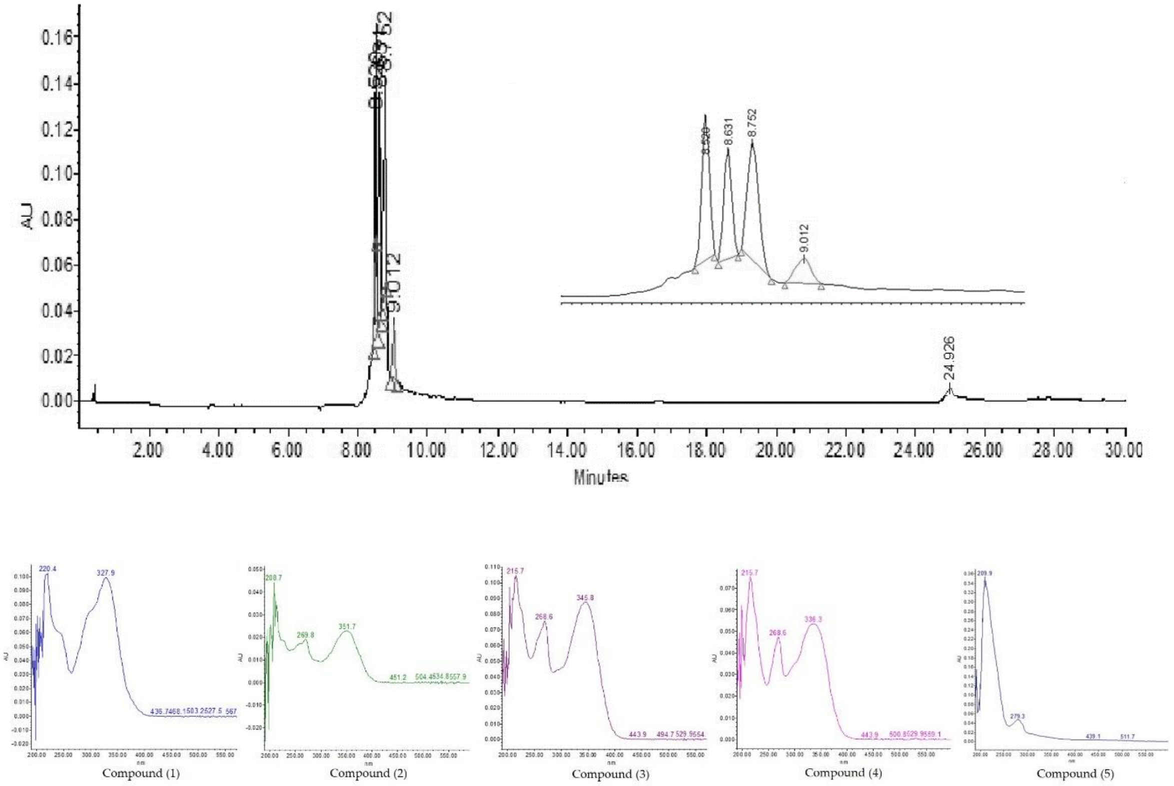

Figure S1: HPLC chromatogram and UV-spectra of the main compounds of hydroalcoholic extracts of **Cp1**

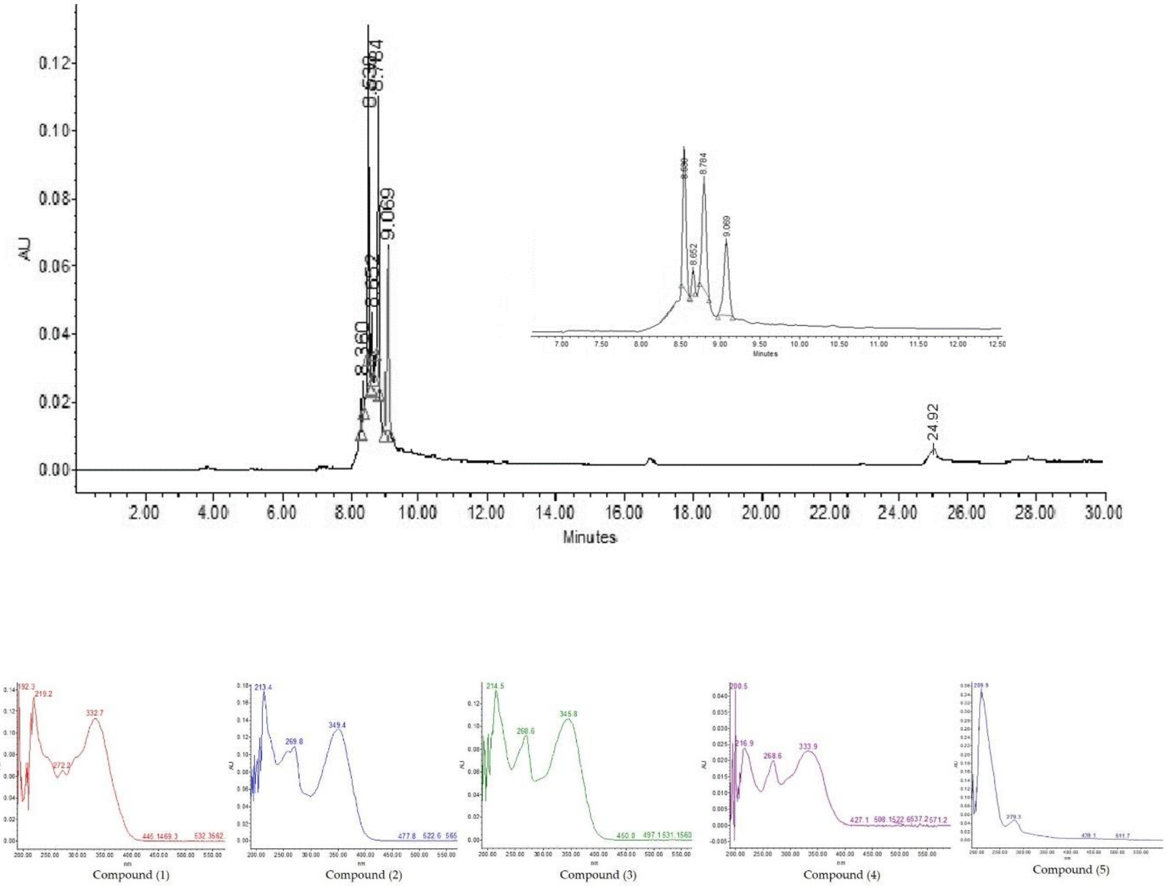

Figure S2: HPLC chromatogram and UV-spectra of the main compounds of hydroalcoholic extracts of **Cp2**

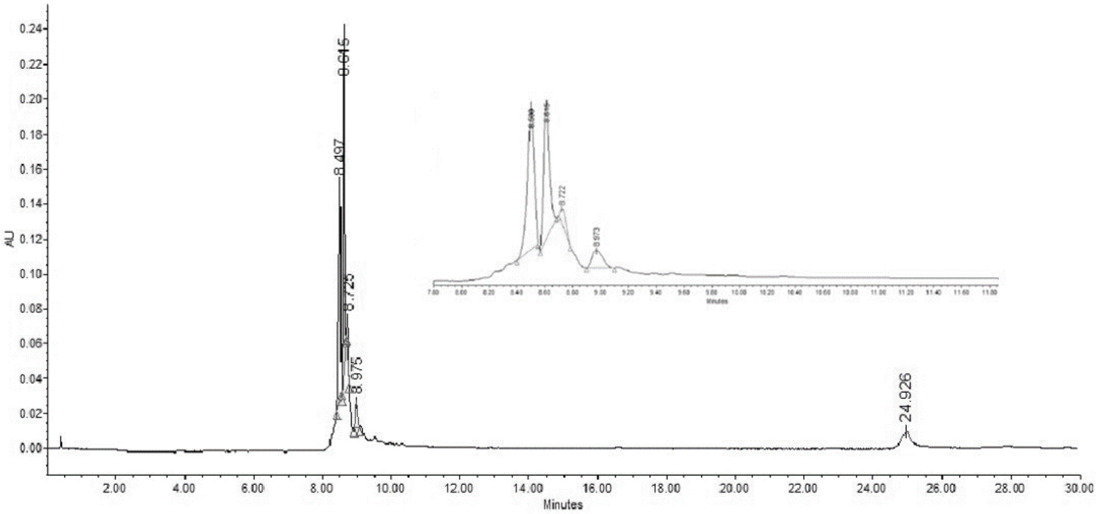

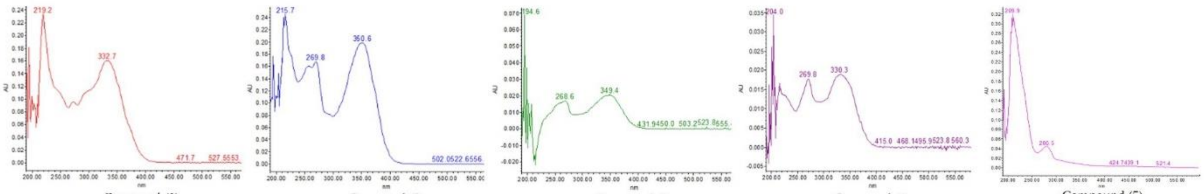

Compound (1) Compound (2) Compound (3) Compound (4) Compound (5)

Figure S3: HPLC chromatogram and UV-spectra of the main compounds of hydroalcoholic extracts of **Cp3**

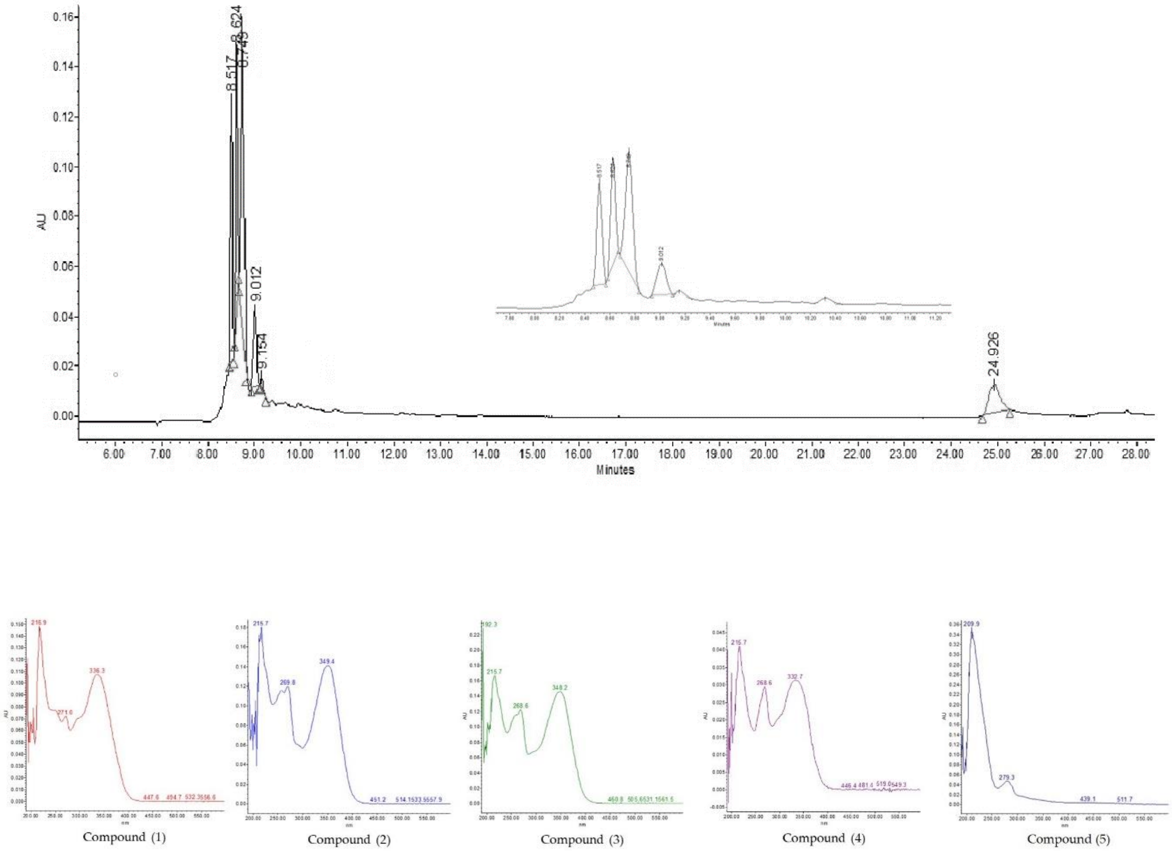

Figure S4: HPLC chromatogram and UV-spectra of the main compounds of hydroalcoholic extracts of **Cp4**

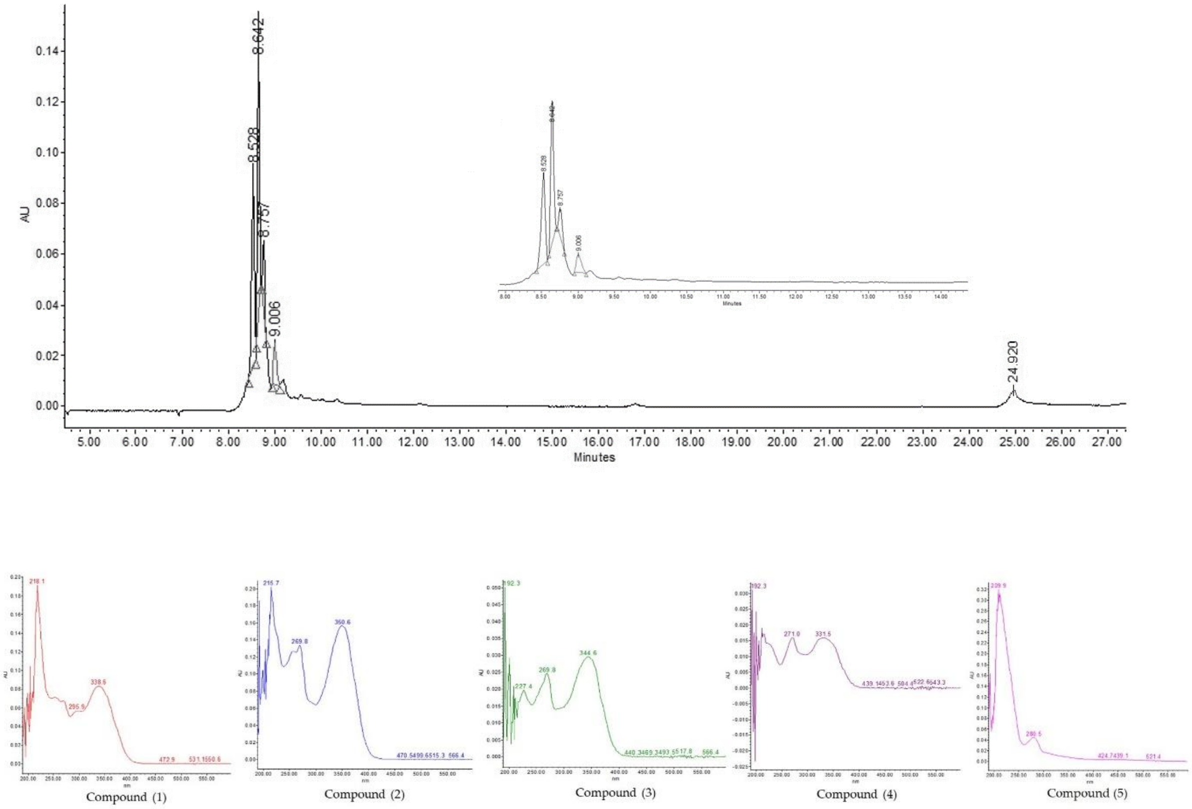

Figure S5: HPLC chromatogram and UV-spectra of the main compounds of hydroalcoholic extracts of **Co5**
